# A novel biomarker Linc00974 interacting with KRT19 promotes proliferation and metastasis in hepatocellular carcinoma

**DOI:** 10.1038/cddis.2014.518

**Published:** 2014-12-04

**Authors:** J Tang, H Zhuo, X Zhang, R Jiang, J Ji, L Deng, X Qian, F Zhang, B Sun

**Affiliations:** 1 Liver Transplantation Center of the First Affiliated Hospital and State Key Laboratory of Reproductive Medicine, Nanjing Medical University, Nanjing, Jiangsu Province, P.R. China

## Abstract

Location-associated long noncoding RNA (lncRNA) was reported to interact with target protein via a *cis-*regulatory process especially for the Flank10kb class lncRNA. Based on this theory, we aimed to explore the regulatory mechanisms of Linc00974 and KRT19 (an lncRNA beyond the Flank10kb class with protein) when we first confirmed the aberrant expression in hepatocellular carcinoma in a previous study. Knockdown of Linc00974 resulted in an inhibition of cell proliferation and invasion with an activation of apoptosis and cell cycle arrest *in vitro*, which was also validated by a subcutaneous and tail vein/intraperitoneal injection xenotransplantation model *in vivo*. We further investigated the interaction pattern of Linc00974 and KRT19. MiR-642 was identified, by acting as the competing endogenous RNA in regulating Linc00974 and KRT19. Linc00974 was increased owing to an abnormal hypomethylation promoter, which induced the upregulation of KRT19 via ceRNA interaction, resulting in the activation of the Notch and TGF-*β* pathways as detected by cDNA microarray. We also discovered Linc00974F-1 stably expressed in the plasma. By the combined analysis of Linc00974F-1 with CYFRA21-1, we found that these joint indicators predicted growth and metastasis of tumor in HCC patients. In conclusion, the combination of Linc00974 and KRT19 may be novel indices for clinical diagnosis of tumor growth and metastasis in HCC, while Linc00974 may become a potential therapeutic target for the prevention of HCC progression.

Hepatocellular carcinoma (HCC) is the sixth most common malignant tumor and the third leading cause of cancer-related death globally.^1^ HCC is characterized by aggressiveness, invasiveness, especially intrahepatically, and frequent recurrence after resection.^2^ Although several approaches have been explored to investigate the potential mechanism of HCC progression, few biomarkers have been identified for dynamic monitoring of invasion or recurrence of HCC.

The long noncoding RNAs (lncRNAs) were reported as a biomarker for predicting survival and metastasis and in the diagnosis of multiple diseases.^3, 4^ Several lncRNAs have been described in liver disease and in liver cancers.^5, 6^ The functional effects of lncRNA have been widely recognized, including regulating gene expression through modulation of chromatin remodeling, controlling of gene transcription, posttranscriptional mRNA processing, protein function or localization, and intercellular signaling.^6, 7, 8^ Mechanisms that have been described for selected lncRNA involved in liver disease include widely diverse functions such as DNA imprinting, X inactivation, DNA demethylation, gene transcription, and generation of other RNA molecules.^9, 10^ Furthermore, several researchers have discovered that lncRNAs were involved in a network that could be modified epigenetically, including methylation, ubiquitination, and miRNA-induced regulation.^10, 11^

The ability to detect lncRNA within the human genome has been facilitated by genomic sequencing and bioinformatics analyses; validation of putative candidate genes is sophisticated due to the various mechanisms described above. The function of most lncRNA implicated in the liver and other diseases remains poorly described. Understanding these functions will be critical to recognizing the contribution of these genes in biological processes involved in hepatic functioning. Bioinformatics analyses recently have reported an underlying method to discover the putative candidate genes in which a ‘Flank10kb' analysis was described.^12^ The novel analysis revealed that >65% of lncRNA genes were located within 10 kb of known, primarily protein-coding genes. They suggested that *cis-*regulatory relationships may exist between lncRNAs and known genes. Additionally, various experimental studies have confirmed that gene-proximal (i.e., Flank10kb class) lncRNAs are likely to regulate their coding genomic neighbor in a *cis-*regulatory or in a *trans-*regulatory manner.^13, 14, 15^ However, CCAT1-L is transcribed specifically in human colorectal cancers from a locus 515 kb upstream of MYC^16^ and has been confirmed to be a *cis-*regulatory gene beyond the Flank10kb class.

According to the bioinformatics analyses above, we were confused with the interaction of location-associated lncRNAs and the protein gene. Thus we consulted several databases, including PubMed, UCSC Genome Browser RNAdb, and fRNAdb. A validated lncRNA named Linc00974 was selected as the candidate lncRNA located upstream of the protein-coding gene KRT19 beyond the Flank10kb class. KRT19 as well as the circulation fragment CYFRA21-1 was defined as HCC progression-associated factor, including predicting metastasis and poor prognosis, while the functional role of Linc00974 in human disease still remains unclear.

In this study, we first aimed to investigate whether Linc00974 was involved in hepatocarcinogenesis or HCC progression. In addition, we attempted to identify the mechanism of interaction of Linc00974 and KRT19, in order to determine whether the Flank10kb-class beyond Linc00974 regulated KRT19 through *cis-* or *trans-*regulatory methods. Finally, we focused on the clinical application in diagnosis or prognostication of Linc00974 by correlation analysis with CYFRA21-1.

## Results

### Linc00974 was upregulated with a high correlation with KRT19 in HCC

**Figure 1:**
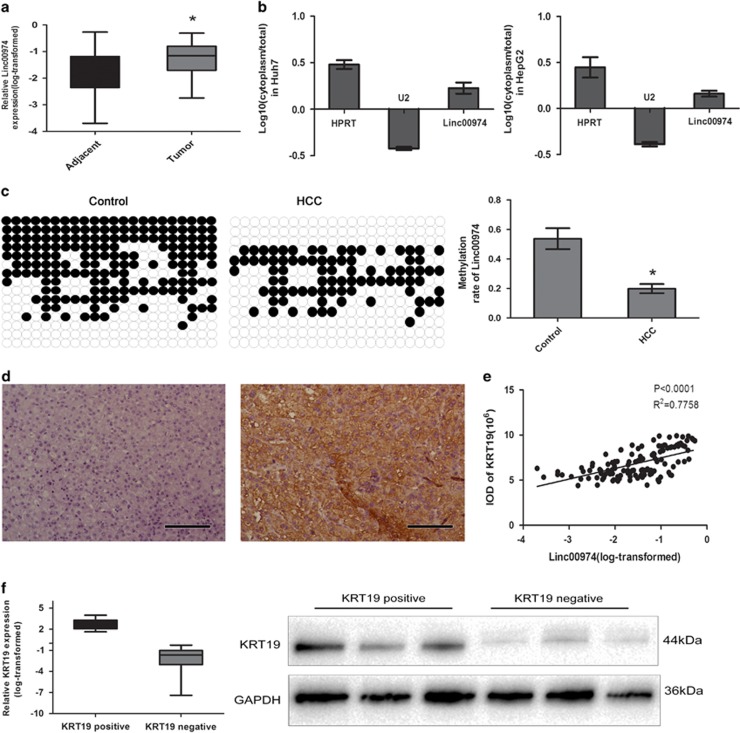
Linc00974 was upregulated with a high correlation with KRT19 in HCC. (**a**) A relatively increased level of Linc00974 was detected in HCC tissues compared with the corresponding adjacent tissues (*n*=150). Data was log-transformed as presented with mean±S.D. (**b**) Subcellular localization investigation indicated that the transcript for Linc00974 was located mainly in the cytoplasm of Huh7 and HepG2 cell lines, according to the results of RT-PCR amplified with separated cytoplasm RNA and nucleus RNA. HPRT was used as the control for cytoplasmic expression and U2 for cytonuclear expression. (**c**) The bisulfite sequencing method was used to detect methylation of CpG island predicted in Linc00974. The methylation level of the Linc00974 promoter was downregulated in tumor tissues compared with the corresponding adjacent tissues. (**d**) Paraffin sections from the above 150 patients were used to examine KRT19 expression; the left indicated a negative stain of KRT19 while the right presented the positive stain. (**e**) Pearson correlation showed a positive correlation between expression levels of KRT19 and Linc00974 with a *P*<0.0001, *R*^2^=0.7753. (**f**) Relative expression level of KRT19 mRNA and protein in HCC samples. Data was log-transformed as presented with mean±S.D. (*P<0.01)

According to the bioinformatics analyses, Linc00974 was located upstream of KRT19, with a spacing of about 25 kb (Supplementary Figure S1A) which was beyond the Flank10kb class. In order to investigate whether Linc00974 correlated with HCC, we first detected the expression level of Linc00974 in tumor tissues and corresponding adjacent tissues. Aberrant increased Linc00974 was detected in tumor tissues, indicating that Linc00974 was involved in the pathogenesis of HCC (Figure 1a).

As Linc00974 has not been reported in human disease, we conducted the experiment to confirm sub-cellular localization, non protein-coding feature, and the expression stability of Linc00974 in liver tissues and cell lines. The transcript for Linc00974 was located primarily in the cytoplasm of Huh7 and HepG2 cell lines according to the results of RT-PCR amplified with separated cytoplasm RNA and nuclear RNA (Figure 1b). The Coding Protein Calculator (http://cpc.cbi.pku.edu.cn/) was used to examine the protein-coding ability of Linc00974. As compared with MEG3, a well-known non-coding RNA Linc00974 was more likely to be the non-coding RNA (Supplementary Figure S1B). Furthermore, we predicted that the second structure of the transcript for Linc00974 indicated a non-coding structure (Supplementary Figure S1C). RACE PCR confirmed that Linc00974 was present and that the full length of the Linc00974 was similar to that determined by RACE analysis in cell lines (Supplementary Figure S1D).

To further explore the embedded mechanism leading to the aberrant expression level of Linc00974, we first investigated the methylation level of Linc00974 in the promoter region bases on the CpG island prediction by using Methyprimer (http://www.urogene.org/methprimer/index.html) and USCS genome bioinformatics (http://genome.ucsc.edu) (Supplementary Figures S4B and D). After detection, we found that the methylation rate in the promoter of Linc00974 was much lower in tumor tissues of HCC patients compared with the corresponding adjacent tumor tissues as the control group (Figure 1c). This might be reasonable for explaining the upregulation of Linc00974 in HCC.

KRT19 was known as a potential biomarker for cholangiocytes, hepatic progenitor cells, and early hepatoblasts, which have been linked with a poor prognosis and metastasis for patients diagnosed with HCC.^17^ We conducted the experiment to detect KRT19 expression in the 150 samples described above by RT-PCR, IHC, and western blotting. Out of the 150 samples, 27 presented a positive stain while 123 were negative (Figure 1d). The integral optical density (IOD) of the stained sections was calculated, and then the correlation of the IOD of KRT19 and the expression level of Linc00974 was analyzed by the Pearson correlation. A positive correlation was obtained with a *P*-value of <0.0001 and *R*^2^=0.7753, indicating a potential positive regulatory effect between Linc00974 and KRT19 (Figure 1e). The mRNA expression level of KRT19 and the representative band of the protein expression level of KRT19 is presented in Figure 1f.

The clinical information was collected to detect whether the aberrant expression of Linc00974 and KRT19 was associated with clinicopathological characteristics. As presented in Table 1, the median value was set as the cutoff to classify the expression level of Linc00974 while KRT19 was divided into positive and negative groups according to IHC staining. The tumor size was grouped using 5 cm as the cutoff level, which was the threshold of indicating small HCCs. Both Linc00974 and KRT19 expression significantly correlated with decreased tumor differentiation grade (*P*<0.001), increased tumor size (*P*<0.001), and metastasis (*P*<0.001).

### Linc00974 promoted cell proliferation and invasion *in vitro*

**Figure 2:**
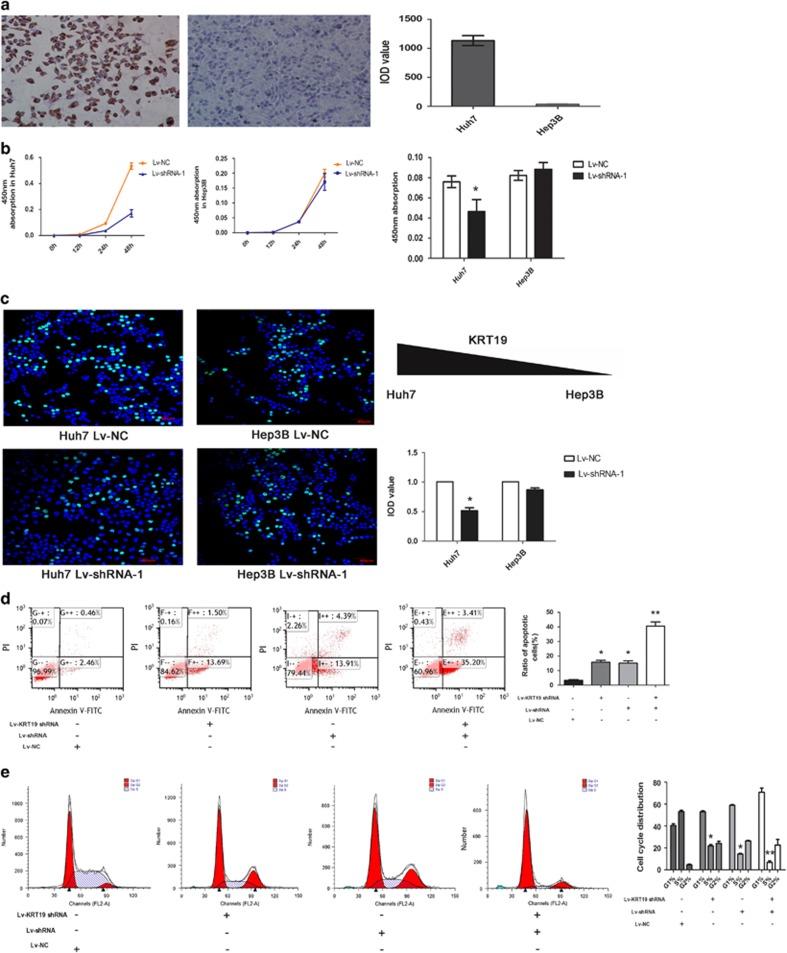
Linc00974 promote cell proliferation in KRT19-positive cells. (**a**) Immunohistochemical analysis was applied to detect KRT19 in HCC cell lines. We selected Huh7 (left panel) as a 100% positive stain while Hep3B (right panel) indicated an absence of KRT19 with a magnification of × 200. (**b**) The CCK8 assay presented showed that a decreased level of Linc00974 inhibited the growth of Huh7, and by contrast, no difference was observed in Hep3B. Absorbance at 450 nm was presented as the mean±SEM. (**c**) The EDU assay confirmed the functional role of Linc00974 in cell proliferation. Stable knockdown of KRT19 reduced the proliferation of Huh-7 after 24 h (*P*<0.01) compared with the negative control while no significant differences in proliferation were found in Hep3B with a magnification of × 200. The IOD value of cells treated with control plasmids was normalized to 100%. (**d**) Cells treated with lncRNA shRNA and KRT19 shRNA followed. Forty-eight hours after treatment, cells were stained and analyzed by flow cytometry. LR, early apoptotic cells; UR, terminal apoptotic cells. (**e**) At 48 h after treatment, cell cycle was analyzed by flow cytometry. The bar chart represents the percentage of cells in G1–G0, S, or G2–M phase. All experiments were performed in triplicate and presented as the mean±S.E.M. (*P<0.05, **P<0.01)

To detect whether Linc00974 could regulate KRT19 in cell physiological function, we first detected the expression of KRT19 in multiple HCC cell lines, and finally selected Huh7 as the KRT19-positive cell and Hep3B as the KRT19-negative cell (Figure 2a). The expression level of Linc00974 was knocked down by two independent shRNA plasmids and was confirmed to be stably downregulated in the two cell lines mentioned above; shRNA-1 was applied to detect the biological significance of Linc00974 on tumor growth and metastasis with better efficiency while KRT19 was also knocked down by shRNA (Supplementary Figures S2A–C). By using the CCK8 assay to determine the functional role of Linc00974 in cell growth (Figure 2b), a decreased level of Linc00974 inhibited the growth of Huh7. By contrast, no difference was observed in Hep3B. Furthermore, we conducted the EDU (5-ethynyl-2′-deoxyuridine) assay to confirm the functional role of Linc00974 in cell proliferation. Stable knockdown of Linc00974 reduced proliferation of Huh-7 after 24 h (*P*<0.01) compared with negative controls, whereas no significant differences in proliferation were found in Hep3B (Figure 2c).

To detect whether the cell apoptosis or cell cycle was affected, the FACS technology was applied. The results revealed that cells treated with either lncRNA shRNA or KRT19 shRNA could induce cell apoptosis. The co-treated cells indicated an apoptosis enhancement (Figure 2d). To further determine the physiological role of Linc00974 in cells cycle, cells were treated with lncRNA shRNA and followed by KRT19 shRNA. After 48 h, cell cycle was analyzed by flow cytometric analysis. Our experiment showed that co-treatment of lncRNA shRNA and KRT19 shRNA could cause remarkable cell cycle arrest (Figure 2e).

**Figure 3:**
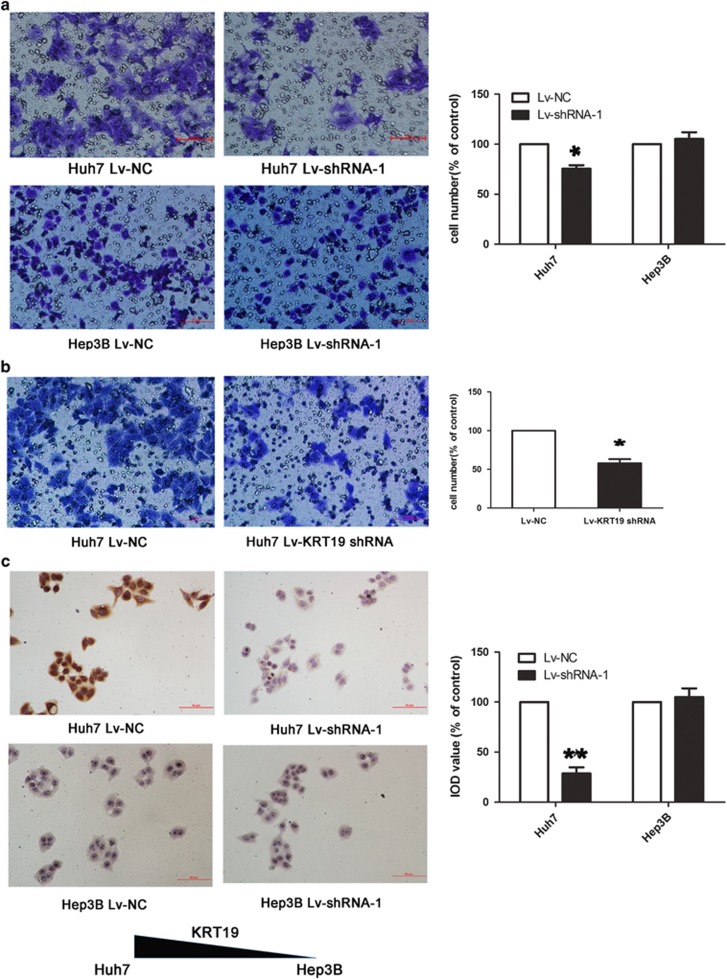
Decreased level of Linc00974 inhibited cell invasion with the downregulation of KRT19 in KRT19-positive cells. (**a**) Cell morphology graph of invasive cells in Huh7 and Hep3B cells after stable transfection of Linc00974 shRNA or negative control. (**b**) Cells were treated with KRT19 shRNA and control shRNA in Huh7. Magnification: × 400. The number of cells treated with control plasmid was normalized to 100%, and data are presented as the mean±S.E.M. based on at least three independent experiments. *P<0.05. (**c**) Immunohistochemical analysis was applied to detect the expression of KRT19 in cells treated with Linc00974 shRNA and control. IOD analysis indicated that Huh7 cells transfected with Linc00974 shRNA had a decreased level of KRT19 while no difference was obtained in Hep3B cells. Magnification: × 400. IOD value of cells treated with control plasmid was normalized to 100%. IOD value was presented as the mean±S.E.M. *Indicates a significant difference compared with the control group (*P*<0.05). **P<0.01

The clinicopathological relevance analysis indicated that Linc00974 might participate in HCC metastasis. To prove this, we conducted the transwell assay to detect the functional role of Linc00974 in cell invasion. The percentage of migrated cells was decreased in Huh7 treated with Linc00974 shRNA while no significant difference was obtained in Hep3B cells (Figure 3a). Furthermore, by knocking down the expression of KRT19 in KRT19-positive cells (Huh7), we found that the migration ability of cells was suppressed comparing with the control (Figure 3b). Based on the results above, we proposed that Linc00974 could inhibit cell proliferation and invasiveness in the presence of KRT19.

We next conducted the experiment to investigate whether knocked down Linc00974 could regulate KRT19 levels in cells by using the IHC assay. The degree of KRT19 dyeing was remarkably decreased in Huh7 cells compared with the control group treated with empty plasmid; however, no alternation was observed in Hep3B, indicating that the abnormal loss expression of Linc00974 could decrease KRT19 (Figure 3c).

### Linc00974 induced an inhibition of tumor growth and metastasis *in vivo*

**Figure 4:**
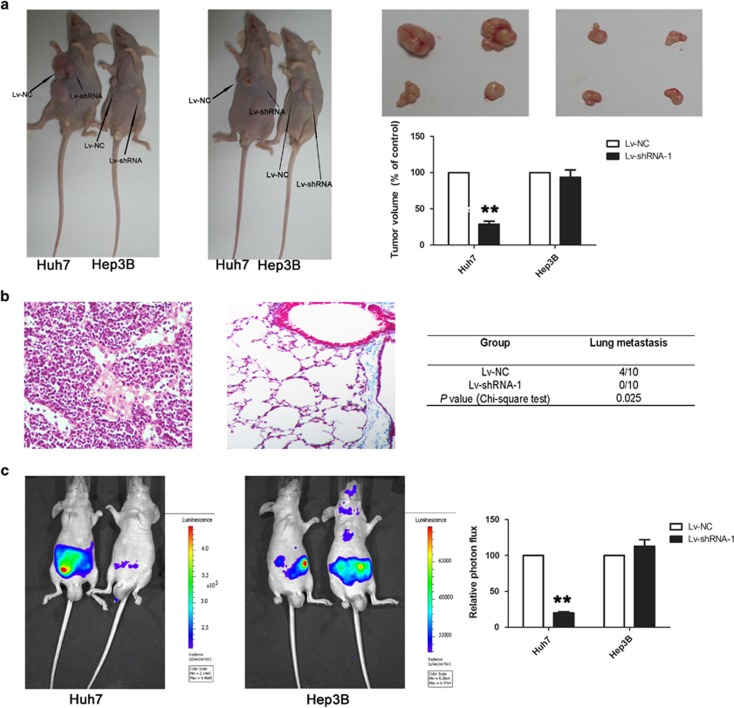
Linc00974 downregulation suppressed tumor growth and metastasis *in vivo*. (**a**) Bilateral axillary of BALB/C nude mice were subcutaneously transplanted with Huh7 or Hep3B stably expressed with Linc00974 shRNA (left axilla) or control (right axilla) (*n*=5). As was indicated by arrows, 4 weeks after implantation, Linc00974 decreased Huh7 attenuated tumor growth in nude mice. The volume of each tumor was calculated as the length × width^2^ × 0.5. The tumor volume of cells treated with controls was normalized to 100%. Data were presented as the mean±S.E.M. *Indicates a significant difference compared with controls (*P*<0.05). (**b**) Representative figures of tail vein xenograft model indicated lung colonization, which was formed in Huh7 cells compared with controls (*n*=10). The number of mice with metastastic foci in the lung was calculated in each group, as presented in the table. Four out of the 10 mice in Huh7 treated with controls presented lung colonization while no metastatic foci were obtained in cells treated with Linc00974 shRNA. The chi-squared test was used to analyze the difference (*P*=0.025). (**c**) An intraperitoneal transplantation vaccination tumor model was applied to detect abdominal metastases. As presented in the left panel, fluorescence intensity was measured using the IVIS Lumina II system. Mice injected withHuh7 resulted in the suppressed metastasis of tumor cells compared with controls; however, no statistically significant difference was obtained in Hep3B. The value of fluorescence intensity in control cells was normalized to 100% data were presented as the mean±S.E.M. *Indicates a significant difference compared with the control group (*P*<0.05), **P<0.01

To determine the effects of Linc00974 on tumorigenesis *in vivo*, nude mice were subcutaneously injected with Huh7 and Hep3B cells stably knocked down for Linc00974 or a control vector in a xenotransplantation model. The left axilla was injected with cells treated with lv-Linc00974 shRNA, and the right side was injected with control cells. We observed that mice injected with Huh7 (absence of Linc00974) showed significantly decreased tumor growth compared with controls; no difference was obtained in Hep3B cells (Figure 4a). Next, we performed a tail vein xenograft model to investigate lung metastases of HCC cell lines. The Huh7 cell line was divided into the Linc00974 knockdown group and the control group with 10 mice in each group. The results showed that some lung colonization was formed in Huh7 cells with the decreasing level of Lin00974, while four mice presented lung colonization at the end of the experiment, and the results were confirmed by histological examination (Figure 4b). To confirm the effect of Linc00974 downregulation on abdominal metastases, we conducted an intraperitoneal transplantation vaccination tumor model with nude mice. In this model, lower expression level of Linc00974 reduced invasion and abdominal metastases in Huh7 cells; however, no reduction was observed in Hep3B according to the photon flux detected (Figure 4c).

We also detected the expression level of KRT19 in tumor samples obtained above. We found that Linc00974 was remarkably decreased in cells treated with shRNA in both Huh7 and Hep3B cell lines. KRT19 was downregulated in Huh7 cells treated with Linc00974 shRNA; however, no dysregulation was found in Hep3B cells (Supplemenray Figures S2D–F).

### Linc00974 affected KRT19 expression by acting as a competing endogenous RNA (ceRNA) interacting with miR-642

**Figure 5:**
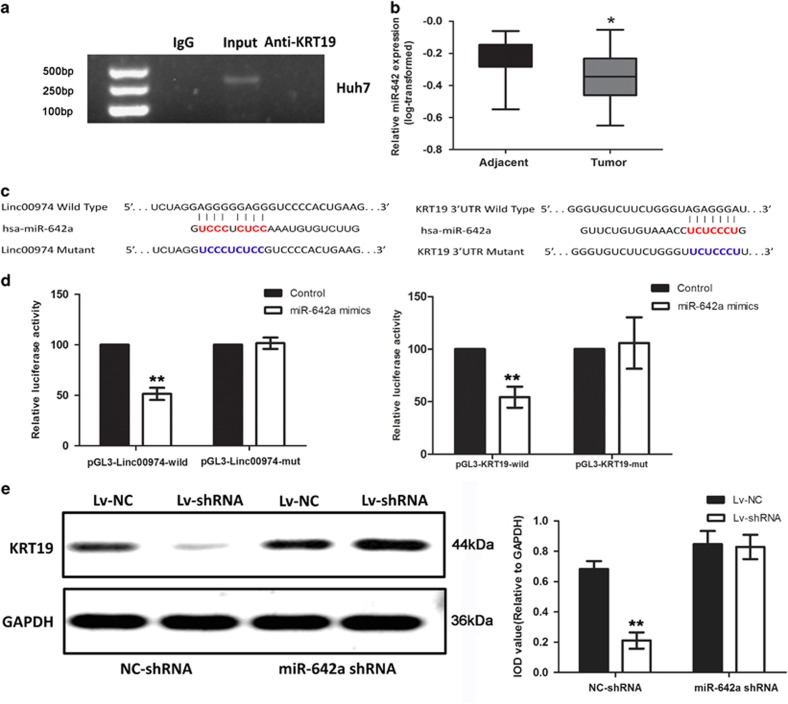
Linc00974 could regulate KRT19 via ceRNA interacting mechanism with the invasion of miR-642. (**a**) Relative RIP experiments were performed using an antibody against KRT19 on extracts from Huh7 with IgG as a negative control. The enrichment of the Linc00974 was normalized to the input. The purified RNA was used for RT-PCR analysis. The results showed that no bands were detected from the RNA in the group with anti-KRT19. (**b**) Relative expression level of miR-642 in clinical samples. Data was log-transformed as presented with mean±S.D. (**c**) Bioinformatics predicted the binding site between the miR-642 with Linc00974 and KRT19, and the mutation types were conducted into the pGL3 plasmid as presented. (**d**) Cells were co-transfected with miR-642a mimics or control, Renilla luciferase vector pRL-SV40 and Linc00974 full length or KRT19 3′UTR luciferase reporters for 48 h. Both firefly and Renilla luciferase activities were measured in the same sample. Firefly luciferase signals were normalized with Renilla luciferase signals. Cells treated with controls of miRNA were normalized to 100%. (**e**) KRT19 protein expression level was detected by western blotting. Cells were treated with Linc00974 shRNA or miR-642a shRNA and the respective control plasmid. IOD was calculated for each band. All tests were performed in triplicate and presented as the mean±S.E.M. *P<0.05, **P<0.01

The important role of Linc00974 in tumorigenesis and metastasis helped to delineate an underlying mechanism of the interaction between Linc00974 and KRT19. As reported, LncRNA could regulate the target protein mainly by directing binding to the target protein. Thus, to further validate the association between Linc00974 and KRT19, we performed an RNA immunoprecipitation (RIP) assay with an antibody against KRT19 on Huh7 cellular extracts. Unfortunately, we did not observe a significantly higher enrichment level of Linc00974 with KRT19 antibody compared with nonspecific IgG control antibody (Figure 5a).

Based on the results above, we concluded that Linc00974 may not directly regulate the expression of KRT19 and that there might be a potential transmitter or ‘bridge-like factor' involved in the network. Besides, according to the subcellular localization of Linc00974, we suspected that Linc00974 might regulate KRT19 through posttranscriptional modification. After detailed consulting with the regulatory mechanism of LncRNA described previously, we proposed that miRNA might participate in the interaction of KRT19 and Linc00974. We conducted the bioinformatics prediction for co-regulation of miRNA on both KRT19 and Linc00974 by applying multiple databases, including BiBi Serv (http://bibiserv.techfak.uni-bielefeld.de/bibi/Tools_RNA_Studio.html), Targetscan (http://www.targetscan.org), PicTar (http://pictar.mdc-berlin.de), and miRbase (http://www.mirbase.org). MiR-642 was selected as the candidate gene (Supplementary Figures S4A and C). We next examined the expression level of miR-642 in clinical samples. We found that miR-642 was downregulated in tumor samples compared with the adjacent tissues (Figure 5b). After analyzing the correlation of the expression level of miR-642 with clinicopathological information, we found that the downregulated miR-642 was acting as an antagonism role of Linc00974 or KRT19 (Table 1). The luciferase reporter gene assay was applied to confirm the binding of miR-642 with Linc09974 and KRT19. Finally, we found that miR-642 could suppress the activity of both the wild type of Linc00974 and KRT19, as presented in Figures 5c and d.

Nevertheless, this was not enough for us to conclude the ceRNA network of miR-642, Linc00974, and KRT19. We next detected the expression of KRT19 by decreasing the level of Linc00974 and miR-642 in Huh7 simultaneously. The result showed that KRT19 was downregulated when Linc00974 was knocked down, whereas this could be reversed when miR-642 was absent (Figure 5e), indicating that Linc00974 may act as a sponge to absorb miR-642, which would normally suppress KRT19.

### NOTCH and TGF-*β* signal pathways were promoted by the upregulation of KRT19 induced by Linc00974

KRT19 was reported as a biomarker for tumor growth or metastasis in HCC;^17^ however, the detailed pathway involved by the abnormal expression of KRT19 still remained unclear. A microarray-based investigation was employed to determine the potential signal pathways. Huh7 cells were grouped by KRT19 stable knockdown, the normal control plasmid, and the mock group. As presented in Supplementary Figure S3A, aberrant expression genes were selected with 4/0.25 as the cutoff, which were regarded as candidate genes for Gene Set Enrichment Analysis. Gene annotation for enrichment indicated that NOTCH and TGF-*β* signal pathways were highly associated with KRT19 downregulation (Supplementary Figure S3B). We next confirmed the progressive activation of genes participating in the two pathways by western blotting. An obviously reduced level of NOTCH1, JAG1, and DTX1 was obtained by the loss of KRT19 in Huh7 cells instead of Hep3B. Meanwhile, transforming growth factor beta receptor 1 (TGFBR1), one of the most crucial factors in the TGF-*β* signaling pathway, as well as the phosphorylation level of SMAD2 and SMAD3, were decreased along with the absence of KRT19 in Huh7, while no difference was observed in Hep3B (Supplementary Figures S3C–F).

### Linc00974 acted as a biomarker in predicting the growth and metastasis of HCC

**Figure 6:**
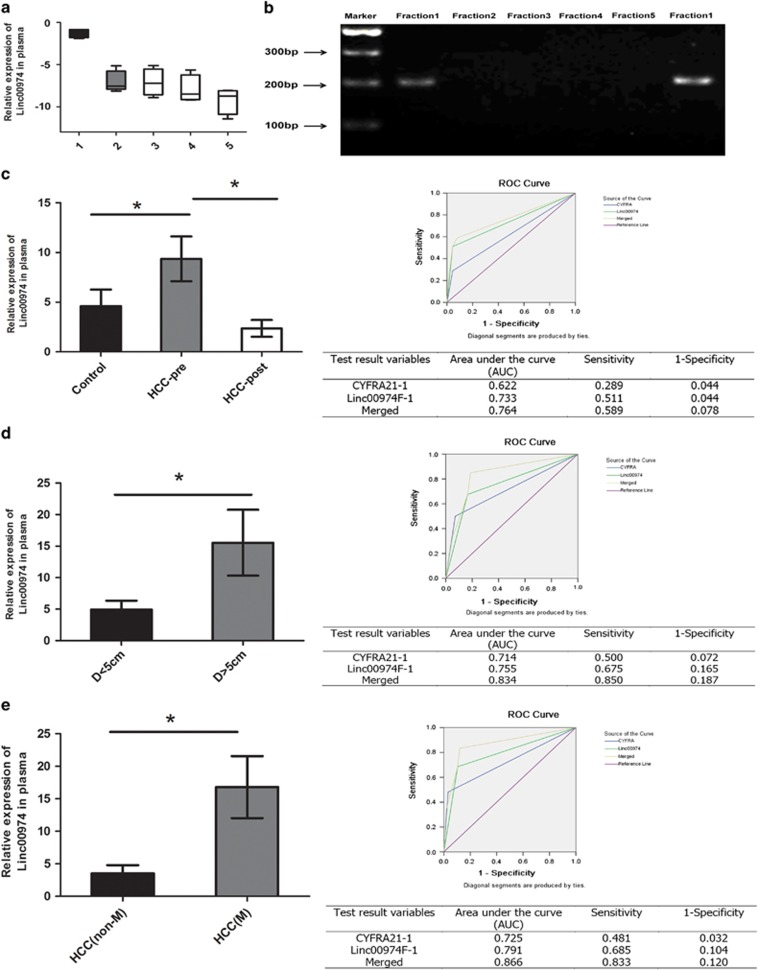
Linc00974 might act as a biomarker in HCC patients. (**a**) Five primers spaced every 500-bp across the complete Linc00974 transcript were designed. qRT-PCR was used to detect the expression of all fractions in HCC plasma samples. The results indicated that fraction1 was the highest expressed in plasma. (**b**) The PCR product was applied for agarose electrophoresis for validation. (**c**) Expression of Linc00974 was detected in patients in whom plasma was obtained from both preoperative and postoperative samples, by comparing with patients free of tumor. ROC curve analysis of merged Linc00974F-1 and CYFRA21-1 was employed to detect the diagnostic efficiency of HCC. Sensitivity and specificity are listed in the left of the curve. (**d** and **e**) Expression of Linc00974 was detected in subgroups grouped by tumor diameter (cutoff: 5 cm) and metastasis. Further ROC curve analysis was used for merged Linc00974F-1 and CYFRA21-1 to predict tumor growth and metastasis in HCC. All experiments are presented as the mean±S.E.M. *Indicates significant difference compared with the control group (*P*<0.05)

Previous reports presented that both miRNA and lncRNA can act as biomarkers for predicting progression and prognosis.^18, 19^ In this study, we were curious about the translation of Linc00974 in clinical life. Thus we attempted to detect the expression pattern of Linc00974 in plasma. Due to the feature of unstable expression level and the easily degradable lncRNA in plasma, we first designed primers for five amplicons (Supplementary Materials) that were found every 500 bp over the complete transcript. We selected fraction1 as the highest expressed amplicon named Linc00974F-1 (Figures 6a and b). Furthermore, the stable expression level of Linc00974F-1 was confirmed by sequencing (Supplementary Figure S4E).

To investigate whether Linc00974F-1 was generated from tumor tissues, we detected the expression level of this fraction in both preoperative and postoperative patients. Linc00974F-1 was remarkably reduced after tumor resection, which suggested that the increased level of Linc00974F-1 in plasma might be secreted by tumor tissues (Figure 6c).

The functional role of Linc00974F-1 in predicting oncogenesis, tumor growth, and metastasis was analyzed. Besides, CYFRA21-1, which is the fraction of KRT19 in plasma, was also employed for the combination of Linc00974F-1. The analysis was based on the results of detecting the expression of Linc00974F-1 and CYFRA21-1 by quantitative real-time PCR (qRT-PCR). The combination analysis of Linc00974F-1 and CYFRA21-1 was calculated by risk score. Receiver operating characteristic (ROC) curve analysis was used to evaluate the value of the predication. The results indicated that the combination of Linc00974F-1 and CYFRA21-1 got the highest area under the curve (AUC) in predicting oncogenesis, tumor growth, and metastasis compared with the AUC calculated for any single factor (Figures 6d and e). According to be expression level of Linc00974F-1 and CYFRA21-1, we clustered HCC patients with 5 cm diameter as the cutoff by hierarchical methods and found that most HCC patients with a tumor diameter <5 cm were clustered in the lower expression level of Linc00974F-1 and CYFRA21-1. Furthermore, we separated patients with metastasis and non-metastasis, and the same cluster method was applied. Most HCC patients with metastasis were clustered in the higher expression levels of Linc00974F-1 and CYFRA21-1 (Supplementary Figure S4F). These results indicated that the combination of Linc00974F-1 and CYFRA21-1 might act as joint biomarkers in predicting the occurrence, growth, or metastasis in HCC.

## Discussion

We aimed to determine the potential regulation pattern between lncRNA and neighbor protein-coding genes based on the multiple molecular mechanisms reported. Their molecular mechanisms of action, function, and contribution to disease pathophysiology are reviewed. LncRNA genes associated with liver diseases have potential roles as biomarkers of disease, prognosis, or therapeutic response as well as direct targets for therapeutic intervention.^20, 21^ Location-associated crosstalk between lncRNA and protein attracted great attention in recent years. To characterize the relationships of lncRNA and protein-coding RNA, researchers have defined three categories of LncRNAs: (1) ‘Flank10kb,' lncRNA genes mapping within 10 kb of a known annotated gene (NCBI RefSeq or UCSC Known Genes) on the same genomic strand; (2) ‘no overlap', lncRNA genes mapping >10 kb from any known gene; and (3) ‘overlap', lncRNAs overlapping a known annotated gene on the same genomic strand.^12^ Based on this theory, they have confirmed that Flank10kb lncRNA candidates that could be joined to their neighboring known genes via complementary DNA (cDNA) or EST contigs should remain annotated as *bona fide* lncRNA genes, if their neighboring genes—despite being included in known gene catalogs—in fact lack the protein-coding capacity. In fact, the Flank10kb lncRNA tended to interact with protein through a *cis-*regulatory process. However, Linc00974, a novel lncRNA confirmed in our study, is first reported in human disease, especially in human HCC. The specific ectopic expression of Linc00974 indicated a positive regulation with KRT19, a protein beyond the Flank10kb class. These unexpected results implied that the regulatory function might be not so absolute in Flank10kb, while the sponge endogenous network might be involved in the potential *cis-*regulatory pathway.

KRT19 by itself is not regarded as an oncogene; it is expressed in nonmalignant cells as well; however, it has been confirmed that KRT19 is a critical player in the proliferation and invasive behavior of some subtypes of HCCs.^22, 23^ The KRT19-associated gene signature showed a strong overlap with that of other previously described more malignant HCC subclasses, such as poor survival or proliferation of HCC subtypes.^24^ The aberrant expression level of NOTCH1, JAG1, and DTX1 was induced by the absence of KRT19 *in vitro* as well as the TGF-*β* signal pathway. The Notch signal pathway was known as an oncogene in various malignant tumors, including HCC.^25^ As we have explored in further investigation, the loss of Linc00974 could result in a suppression of NOTCH signal pathway. The suppression of NOTCH has been confirmed as highly associated with cell apoptosis and cell cycle arrest, which was consistence with our result.^26, 27^ Downregulated NOTCH1 was also reported to be an effective approach to inhibit the proliferation of HCC and could induce tumor formation in mice.^28^ In addition, another signal pathway activated by KRT19 confirmed in our study was also known to participate in tumor invasion and migration. Non-coding RNA could suppress tumor growth and metastasis by targeting TGFBR1 in HCC. Silencing TGFBR1 by small interfering RNA (siRNA) resembled the phenotype resulting from ectopic expression of non-coding RNA.^29^ Our study provided novel, insightful information for the crucial role of KRT19 in the pathogenesis of HCC, especially in tumor growth and metastasis. Thus KRT19 might be a novel biomarker for dividing HCC patients into two groups. The patient-specific treatment target KRT19 may be an open horizon for HCC treatment.

The most important result in our study is that we found the fraction of Linc00974 in the plasma. Furthermore, we combined the fraction with CYFRA21-1, a well-known biomarker for tumor, especially in lung cancer.^30^ CYFRA21-1 acting as the biomarker was not very specific in predicting the HCC-associated index^31^ and thus was not common in clinical diagnosis. The combination results confirmed that the joint indicators of Linc00974F-1 and CYFRA21-1 would be more efficient for predicting growth and metastasis in HCC and could be a potential risk factor for the direction of early intervention. For example, a Linc00974 antagonist might be developed for targeting upregulation of KRT19, which may be involved in tumor growth and metastasis.

In conclusion, we identified a novel Linc00974 in human HCC that was upregulated due to hypomethylation in the promoter region of the tumor. We confirmed a positive regulatory effect between Linc00974 and KRT19, inducing miR-642 acting as the ceRNA. Aberrant expression of Linc00974 increased KRT19 levels, resulting in the activation of both Notch and TGF-*β* signaling pathways and, later, causing the proliferation and invasion of HCC both *in vitro* and *in vivo*. In addition, we discovered a plasma fraction of Linc00974 in the ROC curve analysis by binding Linc00974F-1 and CYFRA21-1, indicating a significant predication of tumor growth and metastasis of HCC. We proposed that the combination of Linc00974 and KRT19 might be a novel index for clinical diagnosis, while Linc00974 could be applied as a potential target for the prevention of HCC progression.

## Materials and Methods

### Patient samples

Study data were obtained from 150 patients who presented between August 2011 and September 2013 at The First Affiliated Hospital of Nanjing Medical University (Nanjing, China). Informed consent for blood and tissue analysis was obtained prior to surgery; the study was approved by our Institutional Ethics Committee. All research was performed in compliance with government policies and the Helsinki Declaration. Experiments were undertaken with the understanding and written consent of each subject. Peripheral blood was collected before surgery. Patients' specimens and related clinicopathological data are summarized in Table 1.

### Quantitative real-time PCR

qRT-PCR was performed to determine the expression levels of Linc00974 and mRNAs of all related genes. For the RNA extraction from cytoplasma and cytonucleus was applied by using the SurePrep Nuclear or Cytoplasmic RNA Purification Kit according to the standard protocols of Thermo Fisher Scientific (Rochester, NY, USA). Total RNA was obtained from tissues using TRIzol reagent as described by the manufacturer (Invitrogen Life Technologies Co, Carlsbad, CA, USA). For mRNA detection, total RNAs (500 ng) were reverse transcribed using the reverse transcription kit (Takara, Tokyo, Japan). GAPDH was used as an internal control. All the primer sequences are shown in Supplementary Table S1. qRT-PCR was performed using ABI Prism 7900HT (Applied Biosystems, Foster City, CA, USA) according to the direction of the reagents. The details are as described previously.^32^

### Western blotting

For western blotting, proteins were extracted from tissues or cultured cells using RIPA buffer containing phenylmethanesulfonylfluoride (Beyotime, Nantong, China). Equal amount of protein loading in each lane was confirmed using the GAPDH antibody. The integrated density of the band was quantified by the ImageJ software (NIH, Bethesda, MD, USA). The details are as described previously.^33^

### Immunohistochemical assay

Sections were deparaffinized and followed by rehydration steps through a graded ethanol series and distilled water and then were treated with 3% H_2_O_2_ in methanol for 30 min to block the endogenous peroxidase activity. The sections were rinsed in phosphate-buffered saline (PBS) twice, 5 min each time, and incubated with 10% normal goat serum for 30 min to block non-specific antibody binding. After washing, the samples were incubated with primary anti-rabbit antibody KRT19 (Zsbio, Beijing, China) at 4 °C overnight, washed in PBS for three times, and then incubated with secondary antibodies. Later, the sections were stained with DAB according to the manufacturer's protocols and mounted and photographed using a digitalized microscope camera (Nikon, Tokyo, Japan).

### Cell proliferation and invasion assay

Cell proliferation was assayed using CCK8 and EDU. (Roche, Basel, Switzerland). Invasion was assessed using the *in vitro* assay, as described previously.^33^

### Luciferase reporter gene assay

The 3′-UTR sequence of KRT19 predicted to interact with miR-642a, and the full length of Linc00974 or a mutated sequence with the predicted target sites were inserted into the pGL3 promoter vector. For reporter assay, cells were plated onto 24-well plates. A Renilla luciferase vector pRL-SV40 (5 ng) was also co-transfected to normalize the differences in transfection efficiency.

### The subcutaneous xenotransplantation model

Cells (1 × 10^7^) stably knocked down for Linc00974 expression in Huh7and Hep3B and control cells (Linc00974-NC) were subcutaneously implanted into the bilateral axillas of three BALB/C nude mice. Tumors were measured every week after implantation, and the volume of each tumor was calculated (length × width^2^ × 0.5). All mice were killed 5 weeks afterwards, and sections of tumor tissues were used to establish orthotopically implanted models according to the protocol reported.^34^

### The metastasis model

BALB/C nude mice underwent anesthesia under ketamine (100 mg/kg, i.p.) and xylazine (20 mg/kg, i.p.). Cells with a stably decreased level of Linc00974 or in the control group were suspended in 200 *μ*l PBS and filtered through a sterile 70-*μ*m nylon mesh filter (BD Falcon, Franklin Lakes, NJ, USA) to form a single cell suspension. Cells were then injected into nude mice through the tail vein to establish peripheral intravascular implanted models (10 in each group). Mice were killed after 5 weeks to observe tumor metastasis in the lung. Lung tissues were examined by H–E staining to evaluate the number of tumors.

In addition, we conducted the intraperitoneal transplantation vaccination tumor model in BALB/C nude mice. In this model system, cells treated with Linc00974 shRNA or controls were injected in the abdominal cavity. The mice were kept in pathogen-free conditions. We monitored abdominal metastasis by the IVIS Lumina II system (Caliper Life Sciences, Hopkinton, MA, USA) week.

### RNA immunoprecipitation

RIP experiments were performed using a Magna RIP RNA-Binding Protein Immunoprecipitation Kit (Millipore, Bedford, MA, USA), according to the manufacturer's instructions. Antibody for RIP assays of KRT19 (Cell Signaling Technology, Beverly, MA, USA) was used as described before. Coprecipitated RNAs were detected by RT-PCR. Gene-specific primers used for detecting Linc00974 are presented in Supplementary Table S1.

### Microarray assay detection and bioinformatics analyses

Stably knocked down KRT-19 in Huh7 and the corresponding empty plasmid of PLL3.7-treated control cells were applied to extract the total RNA from three samples in each group and were amplified and transcribed into fluorescent cDNA. Labeled samples were hybridized to the Human Roche NimbleGen mRNA microarray (Roche). Bioinformatics analyses were conducted using the MAS3.0 system (CapitalBio, Beijing, China) and DAVID Functional Annotation Bioinformatics Microarray Analysis (http://david.abcc.ncifcrf.gov).

### ROC curve analysis and cluster analysis

The upper 95% reference interval of Linc00974F-1 and CYFRA21-1 value in controls was set as the threshold to code the expression level of the corresponding factor for each sample as 0 and 1 in patients. A risk score function (RSF) to predict HCC was defined according to a linear combination of the expression level for the two factors. For example, the RSF for sample *i* using information from the two factors was: RSF_*i*_=∑2*j*−1*Wj*.*sij*. In the above equation, *sij* is the risk score for factor *j* on sample *i*, and *Wj* is the weight of the risk score of factor *j*. The risk score of the two factors was calculated using the weight by the regression coefficient that was derived from the univariate logistic regression analysis of each factor. Samples were ranked according to their RSF and then divided into a high-risk group and a low-risk group. Frequency tables and ROC curves were then used to evaluate the diagnostic effects of the profiling and to find the appropriate cutoff point. The cluster analysis was based on the RSF results. Statistical analysis was performed using STATA 9.2 (Stata Corp., College Station, TX, USA) and presented with the GraphPad Prism 5.0 software (La Jolla, CA, USA). Results were considered statistically significant at *P*<0.05 as repoted before.^35^

### Statistical analysis

The results of qRT-PCR were presented as the median (interquartile interval), and other variables were expressed as the mean (S.E.M.). *χ*^2^ tests and the Student's *t*-test analysis of variance were used to evaluate statistical differences in demographic and clinical characteristics. Pearson correlation analysis was used to analyze the relationship of the expression level of tissues between Linc00974 and KRT19 in HCC patients. Risk score analysis was performed to investigate the effectiveness of the Linc00974F-1 and CYFRA21-1 for prediction. Statistical analysis was performed using STATA 9.2 and presented with the GraphPad prism software. In all cases, *P*<0.05 was considered significant.

## Figures and Tables

**Table 1:** The clinicopathological relevance analysis of Linc00974 and KRT19 expression in HCC patients

	**Linc-MD1**			**KRT19**			**miR-642**		
**Feather**	**Low**	**High**	**P-value**	**Positive**	**Negative**	**P-value**	**Low**	**High**	**P-value**
All cases	75	75		27	123		75	75	
									
*Age, years*			0.288			0.555			0.816
<60	55	49		20	84		51	53	
≥60	20	26		7	39		24	22	
									
*Gender*			0.414			0.357			0.174
Male	66	69		23	112		70	65	
Female	9	6		4	11		5	10	
									
*Differentiation grade*			**0.002**			**0.000**			**0.000**
Well	50	30		0	80		20	60	
Moderate	11	13		1	23		10	14	
Poorly	14	32		26	20		45	1	
									
*Tumor size (cm)*			**0.000**			**0.000**			**0.003**
≤5	49	23		2	70		27	45	
>5	26	52		25	53		48	30	
									
*Tumor number*			0.146			0.677			0.467
Solitary	73	69		26	116		70	72	
Multiple	2	6		1	7		5	3	
									
*Tumor capsular*			0.311			0.712			1.000
Incomplete	1	3		1	3		2	2	
Complete	74	72		26	120		73	73	
									
TNM stage (I:II:III)	55 : 10 : 10	30 : 9 : 36	**0.000**	1 : 1 : 25	84 : 18 : 21	**0.000**	13 : 17 : 45	72 : 2 : 1	**0.000**
									
*Metastasis*			**0.003**			**0.000**			**0.000**
Yes	22	40		26	36		50	12	
No	53	35		1	87		25	63	

Total data from 150 HCC patients were analyzed. For the expression of Linc00974, median expression level was used as the cutoff. Data were analyzed by chi-squared test. *P*-value in bold indicates statistically significant.

## Abbreviations

HCC: hepatocellular carcinoma
lncRNA: long noncoding RNA
qRT-PCR: quantitative real-time PCR
RIP: RNA immunoprecipitation
TGFBR1: transforming growth factor beta receptor 1
ceRNA: competing endogenous RNA
ROC: receiver operating characteristic
IOD: integral optical density.

## References

[bib1] 1Shen Q, Fan J, Yang XR, Tan Y, Zhao W, Xu Y et al. Serum DKK1 as a protein biomarker for the diagnosis of hepatocellular carcinoma: a large-scale, multicentre study. Lancet Oncol 2012; 13: 817–826. 22738799 10.1016/S1470-2045(12)70233-4

[bib2] 2Sahasrabuddhe VV, Gunja MZ, Graubard BI, Trabert B, Schwartz LM, Park Y et al. Nonsteroidal anti-inflammatory drug use, chronic liver disease, and hepatocellular carcinoma. J Natl Cancer Inst 2012; 104: 1808–1814. 23197492 10.1093/jnci/djs452 PMC3514167

[bib3] 3Cesana M, Cacchiarelli D, Legnini I, Santini T, Sthandier O, Chinappi M et al. A long noncoding RNA controls muscle differentiation by functioning as a competing endogenous RNA. Cell 2011; 147: 358–369. 22000014 10.1016/j.cell.2011.09.028 PMC3234495

[bib4] 4Batista PJ, Chang HY. Long noncoding RNAs: cellular address codes in development and disease. Cell 2013; 152: 1298–1307. 23498938 10.1016/j.cell.2013.02.012 PMC3651923

[bib5] 5Xu D, Yang F, Yuan JH, Zhang L, Bi HS, Zhou CC et al. Long noncoding RNAs associated with liver regeneration 1 accelerates hepatocyte proliferation during liver regeneration by activating Wnt/beta-catenin signaling. Hepatology 2013; 58: 739–751. 23483581 10.1002/hep.26361

[bib6] 6Takahashi K, Yan I, Haga H, Patel T. Long non-coding RNA in liver diseases. Hepatology 2014; 60: 744–753. 24493213 10.1002/hep.27043 PMC4110118

[bib7] 7Quagliata L, Matter MS, Piscuoglio S, Arabi L, Ruiz C, Procino A et al. Long noncoding RNA HOTTIP/HOXA13 expression is associated with disease progression and predicts outcome in hepatocellular carcinoma patients. Hepatology 2014; 59: 911–923. 24114970 10.1002/hep.26740 PMC3943759

[bib8] 8Hammerle M, Gutschner T, Uckelmann H, Ozgur S, Fiskin E, Gross M et al. Posttranscriptional destabilization of the liver-specific long noncoding RNA HULC by the IGF2 mRNA-binding protein 1 (IGF2BP1). Hepatology 2013; 58: 1703–1712. 23728852 10.1002/hep.26537

[bib9] 9Tuck AC, Tollervey D. A transcriptome-wide atlas of RNP composition reveals diverse classes of mRNAs and lncRNAs. Cell 2013; 154: 996–1009. 23993093 10.1016/j.cell.2013.07.047 PMC3778888

[bib10] 10Ulitsky I, Bartel DP. lincRNAs: genomics, evolution, and mechanisms. Cell 2013; 154: 26–46. 23827673 10.1016/j.cell.2013.06.020 PMC3924787

[bib11] 11Necsulea A, Soumillon M, Warnefors M, Liechti A, Daish T, Zeller U et al. The evolution of lncRNA repertoires and expression patterns in tetrapods. Nature 2014; 505: 635–640. 24463510 10.1038/nature12943

[bib12] 12Jia H, Osak M, Bogu GK, Stanton LW, Johnson R, Lipovich L. Genome-wide computational identification and manual annotation of human long noncoding RNA genes. RNA 2010; 16: 1478–1487. 20587619 10.1261/rna.1951310 PMC2905748

[bib13] 13Rinn JL, Kertesz M, Wang JK, Squazzo SL, Xu X, Brugmann SA et al. Functional demarcation of active and silent chromatin domains in human HOX loci by noncoding RNAs. Cell 2007; 129: 1311–1323. 17604720 10.1016/j.cell.2007.05.022 PMC2084369

[bib14] 14Faghihi MA, Modarresi F, Khalil AM, Wood DE, Sahagan BG, Morgan TE et al. Expression of a noncoding RNA is elevated in Alzheimer's disease and drives rapid feed-forward regulation of beta-secretase. Nat Med 2008; 14: 723–730. 18587408 10.1038/nm1784 PMC2826895

[bib15] 15Yin Z, Guan D, Fan Q, Su J, Zheng W, Ma W et al. lncRNA expression signatures in response to enterovirus 71 infection. Biochem Biophys Res Commun 2013; 430: 629–633. 23220233 10.1016/j.bbrc.2012.11.101 PMC7092842

[bib16] 16Xiang JF, Yin QF, Chen T, Zhang Y, Zhang XO, Wu Z et al. Human colorectal cancer-specific CCAT1-L lncRNA regulates long-range chromatin interactions at the MYC locus. Cell Res 2014; 24: 513–531. 24662484 10.1038/cr.2014.35 PMC4011346

[bib17] 17Govaere O, Komuta M, Berkers J, Spee B, Janssen C, de Luca F et al. Keratin 19: a key role player in the invasion of human hepatocellular carcinomas. Gut 2014; 63: 674–685. 23958557 10.1136/gutjnl-2012-304351 PMC3963546

[bib18] 18Zhang JX, Song W, Chen ZH, Wei JH, Liao YJ, Lei J et al. Prognostic and predictive value of a microRNA signature in stage II colon cancer: a microRNA expression analysis. Lancet Oncol 2013; 14: 1295–1306. 24239208 10.1016/S1470-2045(13)70491-1

[bib19] 19Kumarswamy R, Bauters C, Volkmann I, Maury F, Fetisch J, Holzmann A et al. The circulating long non-coding RNA LIPCAR predicts survival in heart failure patients. Circ Res 2014; 114: 1569–1575. 24663402 10.1161/CIRCRESAHA.114.303915

[bib20] 20Younger ST, Rinn JL. 'Lnc'-ing enhancers to MYC regulation. Cell Res 2014; 24: 643–644. 24777251 10.1038/cr.2014.54 PMC4042177

[bib21] 21Huang JL, Zheng L, Hu YW, Wang Q. Characteristics of long non-coding RNA and its relation to hepatocellular carcinoma. Carcinogenesis 2014; 35: 507–514. 24296588 10.1093/carcin/bgt405

[bib22] 22Ju JH, Yang W, Lee KM, Oh S, Nam K, Shim S et al. Regulation of cell proliferation and migration by keratin19-induced nuclear import of early growth response-1 in breast cancer cells. Clin Cancer Res 2013; 19: 4335–4346. 23833298 10.1158/1078-0432.CCR-12-3295

[bib23] 23Kim H, Choi GH, Na DC, Ahn EY, Kim GI, Lee JE et al. Human hepatocellular carcinomas with "Stemness"-related marker expression: keratin 19 expression and a poor prognosis. Hepatology 2011; 54: 1707–1717. 22045674 10.1002/hep.24559

[bib24] 24Lee JS, Chu IS, Heo J, Calvisi DF, Sun Z, Roskams T et al. Classification and prediction of survival in hepatocellular carcinoma by gene expression profiling. Hepatology 2004; 40: 667–676. 15349906 10.1002/hep.20375

[bib25] 25Luo J, Zhou H, Wang F, Xia X, Sun Q, Wang R et al. The hepatitis B virus X protein downregulates NF-kappaB signaling pathways through decreasing the Notch signaling pathway in HBx-transformed L02 cells. Int J Oncol 2013; 42: 1636–1643. 23450368 10.3892/ijo.2013.1842

[bib26] 26Dai Y, Wilson G, Huang B, Peng M, Teng G, Zhang D et al. Silencing of Jagged1 inhibits cell growth and invasion in colorectal cancer. Cell Death Dis 2014; 5: e1170. 24722295 10.1038/cddis.2014.137 PMC5424114

[bib27] 27Su BH, Qu J, Song M, Huang XY, Hu XM, Xie J et al. NOTCH1 signaling contributes to cell growth, anti-apoptosis and metastasis in salivary adenoid cystic carcinoma. Oncotarget 2014; 5: 6885–6895. 25149541 10.18632/oncotarget.2321 PMC4196170

[bib28] 28Villanueva A, Alsinet C, Yanger K, Hoshida Y, Zong Y, Toffanin S et al. Notch signaling is activated in human hepatocellular carcinoma and induces tumor formation in mice. Gastroenterology 2012; 143: 1660–1669 e1667. 22974708 10.1053/j.gastro.2012.09.002 PMC3505826

[bib29] 29Yang H, Fang F, Chang R, Yang L. MicroRNA-140-5p suppresses tumor growth and metastasis by targeting transforming growth factor beta receptor 1 and fibroblast growth factor 9 in hepatocellular carcinoma. Hepatology 2013; 58: 205–217. 23401231 10.1002/hep.26315

[bib30] 30Sun N, Chen Z, Tan F, Zhang B, Yao R, Zhou C et al. Isocitrate dehydrogenase 1 is a novel plasma biomarker for the diagnosis of non-small cell lung cancer. Clin Cancer Res 2013; 19: 5136–5145. 24046070 10.1158/1078-0432.CCR-13-0046

[bib31] 31Uenishi T, Kubo S, Hirohashi K, Tanaka H, Shuto T, Yamamoto T et al. Cytokeratin-19 fragments in serum (CYFRA 21-1) as a marker in primary liver cancer. Br J Cancer 2003; 88: 1894–1899. 12799633 10.1038/sj.bjc.6601026 PMC2741125

[bib32] 32Kim K, Jutooru I, Chadalapaka G, Johnson G, Frank J, Burghardt R et al. HOTAIR is a negative prognostic factor and exhibits pro-oncogenic activity in pancreatic cancer. Oncogene 2013; 32: 1616–1625. 22614017 10.1038/onc.2012.193 PMC3484248

[bib33] 33Tang W, Qin J, Tang J, Zhang H, Zhou Z, Li B et al. Aberrant reduction of MiR-141 increased CD47/CUL3 in Hirschsprung's disease. Cell Physiol Biochem 2013; 32: 1655–1667. 24334875 10.1159/000356601

[bib34] 34Yuan JH, Yang F, Wang F, Ma JZ, Guo YJ, Tao QF et al. A long noncoding RNA activated by TGF-beta promotes the invasion-metastasis cascade in hepatocellular carcinoma. Cancer Cell 2014; 25: 666–681. 24768205 10.1016/j.ccr.2014.03.010

[bib35] 35Liu R, Chen X, Du Y, Yao W, Shen L, Wang C et al. Serum microRNA expression profile as a biomarker in the diagnosis and prognosis of pancreatic cancer. Clin Chem 2012; 58: 610–618. 22194634 10.1373/clinchem.2011.172767

