# Supplementary material for: A novel biomarker Linc00974 interacting with KRT19 promotes proliferation and metastasis in hepatocellular carcinoma
Source: Cell Death Dis. 2014 Dec 4;5(12):e1549–. doi: 10.1038/cddis.2014.518 (PMC4649834; doi:10.1038/cddis.2014.518)
Supplement: Supplementary Information [file cddis2014518x1.pdf]

Contents:

Supplement meterials: Sequencing information and Supplementary figure legends

Supplementary Figure1

Supplementary Figure2

Supplementary Figure3

Supplementary Figure4

Supplementary Table1: Primer information

Supplementary materials

Detailed sequence of five Linc00974 fragments detection in serum samples (Amplification sequence was highlighted)

CTCTTGGCTTCAGGCTCTGAAGCACTCCCCA**TCTCAGG**CCCAAACAGGAATAAAATCAGTTCCTTTCTCT
GTTCCTCTTACTAGACTGAGATCCACTGGGATTGTCTGAGGGAATTCTTGTGAAACCAGAAAGAAAGTTC
AAGAAATGATGCAACAACAACAAAATAAAACCCAGGAGAAATAGTACGACAAGAAGTTAAAAGATCGTGA
GGAGAGCCCTGCAGTGGAGGAGGAGAACCTGTTGGCCAGGAACTGCCAACAGCTTTTGTTCTGGATAAGC
CCCTCGTGGAGGAGGACAAGGCAAGAAGATGGGTCTTCATCCTAACCCACGGAAGCAGCGCTGGACTGAG
AAGCACCACCTGCGAAACTCCCAGCCCCACTGAGTAACCTCCAGGGCAGGGGCATGTCCCTGCTTACGAG

Forward primer **TCTCAGGCCCAAACAGGAAT**
Reverse primer **CAGGGCTCTCCTCACGATCT**
190nt

CTCCCACCAGCCCGTCACACCCCCCCACCCACCTCAGAGGCTCCTGCCCCTGACCTGAGTGAGCTGTGTC
TGAGTTGGGTCCCTCCTCCCTCCAAGGAGTGTGCTCTATGGACAACAGCCTCTATCCTCATTTGTCCCTT
CCTCCTAGCGCCTCCTTCTGCACCCCTGACCCCGCTCCATGCTGTCACCTCCTCTGTATGGCAGCTGGGC
CTCGTCTGTCCTGGGACGGTGGCAAGCATGTACAGAAGTCATCTTCCTTATTTCTCACTACCCCCAGCTT
CCCAGATGGGAAACTGAGGCTCAGAGAAATGACATAACTCAGCTGACAAAGGATCTGAGTGGGTCTGACC
CGCAAGCCCACCCTCTTCACCTCATGCTGTCTAAAACAATTACAGACACCTCCCACGAGCAGGAAAGCTC

Forward primer **GTGCTCTATGGACAACAGCCT**
Reverse primer **CCATACAGAGGAGGTGACAGC**
101nt

TGGAAATGTCTTCTTGGAAAGCAAGCAGCTACCCATGAACAGACTTCTCAAAGCTTTTGGCCTTGGCCTC
CCAAGGTGCTGGGACTACAGGCGTGGGTCACTGCACCTGGCCAAAGCTTTTGTTTGCTTAAAGTGCTTTG
TAAGTTTATAAGAGGAGGCTACAGAACCCAGAAATCACCTATGGATTTGACCACGAGATGCGCTGCTGAA
TTCATGGTGGGAAATGCTGATGATCTCTGATGGAGGCTGCTGGGTGGCTGCTGCCCTCACCCTGACCTGT
CCTCCCCAGCTGGACGGGGGACTTCTAGGAGGGGGAGGGTCCCCACTGAAGATGGACACATGTCCTGTCA
GAGTTTCTGAGAAGCTCCAGCCCTTAGGCAGAATGGGACAAGGAGAAACAGACTTGGGTCTGTACTCGGG

Forward primer **AGCAAGCAGCTACCCATGAA**
Reverse primer **GCGCATCTCGTGGTCAAATC**
184nt

ACTGCAGAAGCCGAGCATGAGGAGTTGTGGGGACAGTGCCCCTCCTTCATCCCCACCTCCCAGCCCCCCA

GTATCTCCTTCTCCCCCTGATCCTTCCCTTGATCAGGCCAGGGCCCTGTCATTGTCCCAACAAATGCCTA
CCGCCAGTTCATCGCACCTTGTTTTCTCTGATGTCCTCGCAATCACACACCTTGTGCCCCTGAGCTGGGT
ACACGATCCTTTGCAATGTGTGCTACGAGAACTGTATTGCTAATAATAAAGACAAAATTTAATGACTCCA
CTGGCTGAGCTTCTGGTAGGTCCCAGGCACGTTAGACAGACTATTTCATTAATTCTCACCTCGGCAATTT
GAGGAAGGTCTTGCTGTCTCAGTATCCTGAGTCTTAGACAGGTTCAATGACTTGCACAAGGTCACATAGC

Forward primer **GAAGCCGAGCATGAGGAGTT**
Reverse primer **TGAACTGGCGGTAGGCATTT**
145nt

TTTTAAGTGACAGGGCCTGGCTGCCTTGGCTCTGAAGTGAGTTCTCTTTGCAACAACCTACCCAGCTGAG
CCCCTCCTTTGTTTAGAGAAGGCTTCTCCTCTGAGCTGGGAAGATCCAACTTTGACCCTCAATTCTCAGA
TGACAGTGGTTCAGTCAGGCAGGAACTAGCTGTACTAACATGTAAACAAGTCACTTCTGCAAGGAGAGGC
AGAGGCCAGTGCTTGCGCGAGCGTGACTGATAAAGGAATGGGGCGAGGGCTGAGGACTCTCTGGCTCTAC
CGATTTATGATACTGAAAGTCATCAATCTTAAAGAGAGATCTGCATTCCCCAAGCTTTCTTCTCCCTGAG
GGGCCTCTCCTATCTCTCGCCAATATTGTGGCTCCCCTCAGCCGCAGGGGCAATCTTTCTGGTTTTCTCT
TAGGTCCAAACACGAACCAAATAAAATCCTCTTTTCCCTTC

Forward primer **TGACAGTGGTTCAGTCAGGC**
Reverse primer **CCCTCGCCCCATTCCTTTAT**
119nt

Supplementary Figure legends

Supplementary Figure1. Detailed information for Linc00974.

A: The relative physical location of Linc00974 and KRT19 obtained from PubMed database. B: Coding Protein Calculator (http://cpc.cbi.pku.edu.cn/) was employed to examine the protein coding ability of Linc00974. MEG3 was regarded as a positive control. C: Second structure of Linc00974. D: 5’RACE PCR and 3’RACE PCR was used to detected the full length of Linc00974 in cells.

Supplementary Figure2. Expression level of Linc00974 and KRT19 in vitro and in vivo.

A, B: Huh7 and Hep3B were transfected with Linc00974 shRNA or control. Relative expression level of Linc00974 was confirmed by quantitative real time polymerase chain reaction (qRT-PCR). Data was presented as Mean ± SEM. * indicates significant difference compared with control group (P<0.05). C: Huh7 cells were treated with KRT19 shRNA or control. Next, we applied a qRT-PCR assay to detect the expression of KRT19 in Huh7 cells. D: Relative expression level of Linc00974 and KRT19 in tumor tissues obtained from xenotransplantation The mRNA expression data was log-transformed. Data was presented as Mean ± SEM. * indicates significant difference compared with control group (P<0.05).

Supplementary Figure3. Microarray investigation indicated the Notch and TGF-β signal pathway was activated by the absence of KRT19

A: Scatter plot results presented the different gene expression with the cutoff value set at 4 / 0.25, by comparing the KRT19 knockdown group with the control group in Huh7 cells. B: Gene annotation for enrichment of the candidate genes. C, D: Western blot was applied to confirm whether the aberrant suspension of Notch and TGF-β signal pathway were induced by the loss of Linc00974. NOTCH1, JAG1, and DTX1 were selected to determine the aberrant expression of Notch signal pathway, phosphorylated SMAD2 (Ser467) or SMAD3(Ser423/425) was the representative for TGF-β signal pathway. GAPDH was used as a loading control. E, F: Integral optical density (IOD) was calculated for each band. Data were presented as the mean ± SEM. * indicates P<0.05

Supplementary Figure4. Bioinformatics analysis and Cluster analysis

A, C BiBi Serv (http://bibiserv.techfak.uni-bielefeld.de/bibi/Tools_RNA_Studio.html) database was used for prediction the binding site of Linc00974 and miR-642. The result indicated a series of matched sequences with the minimum free energy (MFE) value -28.0 kcal/mol. Targetscan was applied for prediction the binding site of KRT19 and miR-642. B, D: CpG Island of Linc00974 promoter region was predicted by using

Methyprimer (http://www.urogene.org/methprimer/index.html) and USCS genome broinformatics (http://genome.ucsc.edu). E: Sanger sequencing was used to detect the PCR product of Linc00974F-1. F: The unsupervised cluster method was performed to analyze the differential expression of Linc00974F-1 and CYFRA21-1 between the HCC subgroups (Tumor volume and metastasis). The dendrogram generated showed a clear separation tumor volume grouped with 5cm as cutoff and the subgroup of metastasis on the basis of the two factors signature after the cluster analysis.

Supplementary Table 1. Oligonucleotide Sequences

Gene	Sequence	
Linc00974	Forward	5'- TCTAACGTGCCTGGGACCTA-3'
	Reverse	5'- AAATGCCTACCGCCAGTTCA-3'
U2 snRNA	Forward	5'-TTGGCTAAGATCAAGTGT-3'
	Reverse	5'-GAGCAAGCTCCTATTCC-3'
HPRT mRNA	Forward	5'-ATACAAAGCCTAAGATGAG-3'
	Reverse	5'-GATAAGCGACAATCTACC-3'
KRT19	Forward	5'- ACCAAGTTTGAGACGGAACAG -3'
	Reverse	5'- CCCTCAGCGTACTGATTTCCT -3'
ShRNA-Linc00974-1	5’-CACCGCTCAGAGAAATGACATAACTCGAAAGTTATGTCATTTCTCTGAGC-3’	
ShRNA-Linc00974-2	5'-CACCGCTACCCATGAACAGACTTCTCGAAAGAAGTCTGTTCATGGGTAGC-3'	
Linc00974 RIP primer	Forward	5'- TCACTTCAGAGCCAAGGCAG-3'
	Reverse	5'- AAATGCCTACCGCCAGTTCA-3'
ShRNA-KRT19	5’-CACCGGAAGACACACTGGCAGAAACCGAAGTTTCTGCCAGTGTGTCTTCC-3’	

GAPDH	Forward	5'- GGCATCTTGGGCTACACT-3'
	Reverse	5'- GCCGAGTTGGGATAGGG-3'
Methylation primer	Forward	5'- AGTTTGTTGTTGGTTATTTTTTGTG-3'
	Reverse	5'- AACTACCACCCTAAACCTCCATTA-3'

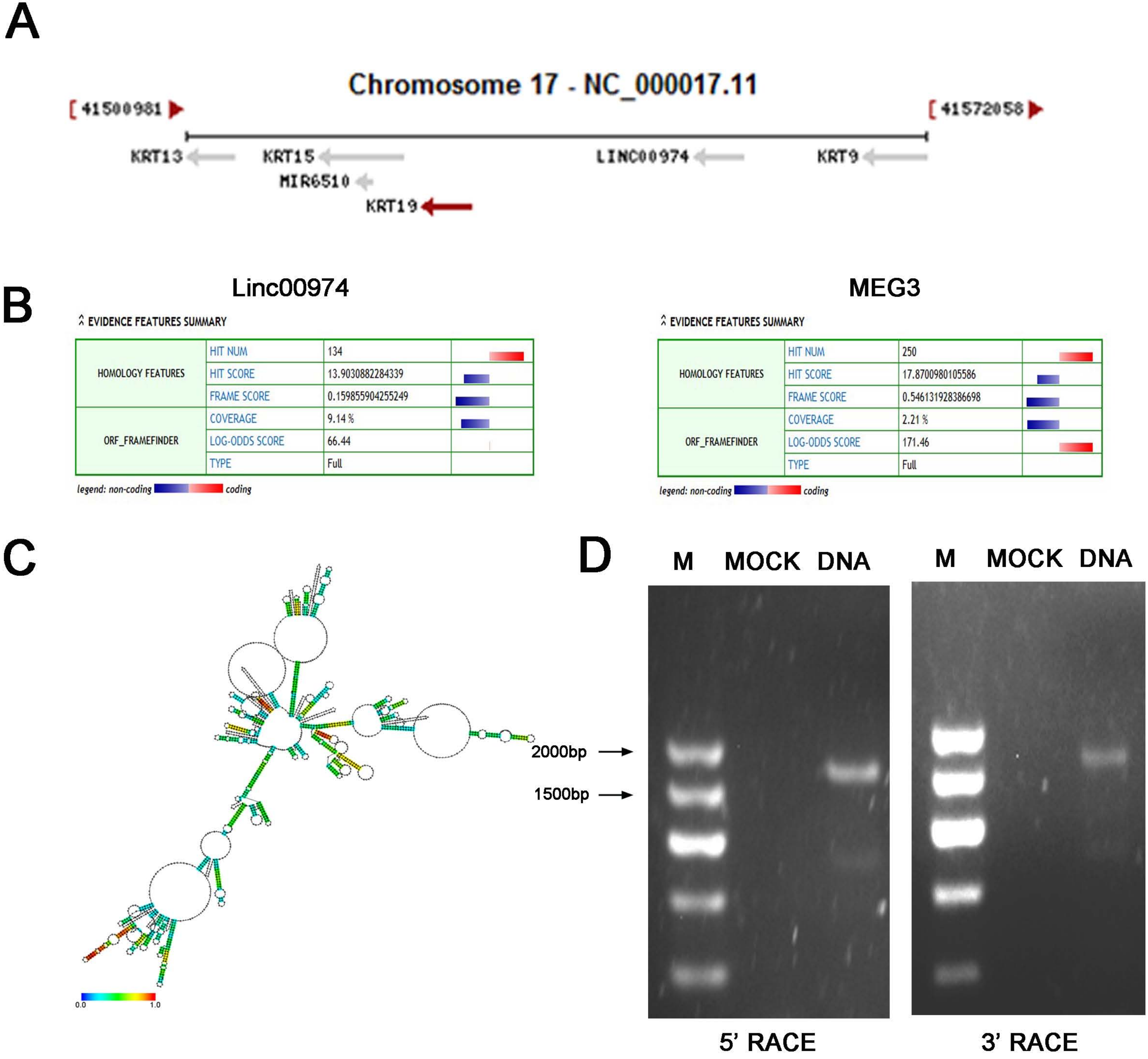
A
Chromosome 17 - NC_000017.11
41500981
41572058
KRT13
KRT15
MIR6510
KRT19
LINC00974
KRT9
B
Linc00974
EVIDENCE FEATURES SUMMARY
HOMOLOGY FEATURES
HIT NUM 134
HIT SCORE 13.9030882284339
FRAME SCORE 0.159855904255249
ORF_FRAMEFINDER
COVERAGE 9.14 %
LOG-ODDS SCORE 66.44
TYPE Full
legend: non-coding coding
MEG3
EVIDENCE FEATURES SUMMARY
HOMOLOGY FEATURES
HIT NUM 250
HIT SCORE 17.8700980105586
FRAME SCORE 0.546131928386698
ORF_FRAMEFINDER
COVERAGE 2.21 %
LOG-ODDS SCORE 171.46
TYPE Full
legend: non-coding coding
C
0.0
1.0
D
M MOCK DNA
M MOCK DNA
2000bp
1500bp
5' RACE
3' RACE

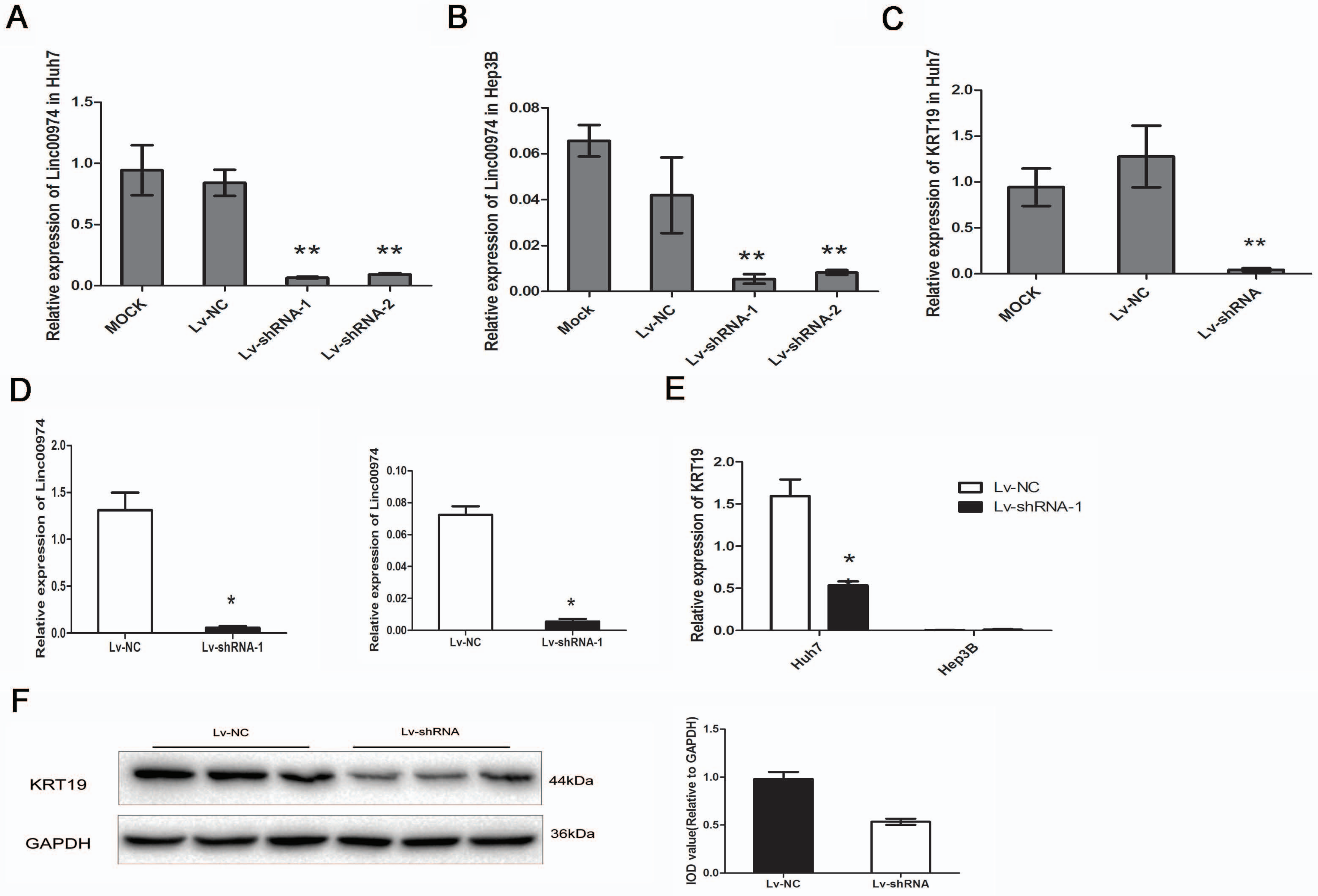

A
Relative expression of Linc00974 in Huh7
MOCK
Lv-NC
Lv-shRNA-1
Lv-shRNA-2
B
Relative expression of Linc00974 in Hep3B
Mock
Lv-NC
Lv-shRNA-1
Lv-shRNA-2
C
Relative expression of KRT19 in Huh7
MOCK
Lv-NC
Lv-shRNA
D
Relative expression of Linc00974
Lv-NC
Lv-shRNA-1
E
Relative expression of KRT19
Lv-NC
Lv-shRNA-1
Huh7
Hep3B
F
Lv-NC
Lv-shRNA
KRT19
GAPDH
44kDa
36kDa
IOD value(Relative to GAPDH)

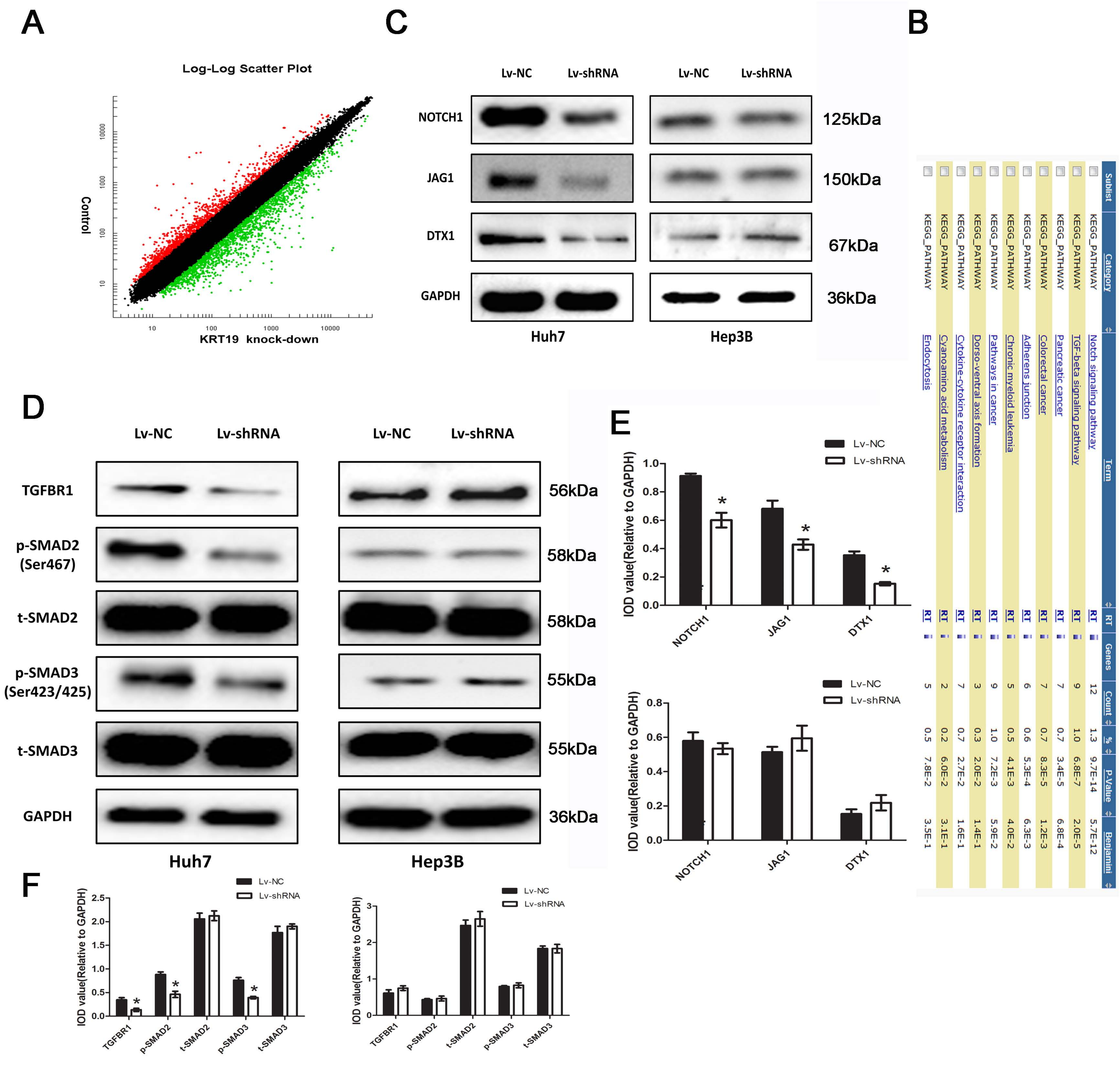
A
Log-Log Scatter Plot
Control
KRT19 knock-down
10
100
1000
10000
C
Lv-NC
Lv-shRNA
Lv-NC
Lv-shRNA
NOTCH1
JAG1
DTX1
GAPDH
125kDa
150kDa
67kDa
36kDa
Huh7
Hep3B
B
Sublist
Category
Term
RT
Genes
Count
%
P-Value
Benjamini
KEGG_PATHWAY Notch signaling pathway RT 12 1.3 9.7E-14 5.7E-12
KEGG_PATHWAY TGF-beta signaling pathway RT 9 1.0 6.8E-7 2.0E-5
KEGG_PATHWAY Pancreatic cancer RT 7 0.7 3.4E-5 6.8E-4
KEGG_PATHWAY Colorectal cancer RT 7 0.7 8.3E-5 1.2E-3
KEGG_PATHWAY Adherens junction RT 6 0.6 5.3E-4 6.3E-3
KEGG_PATHWAY Chronic myeloid leukemia RT 5 0.5 4.1E-3 4.0E-2
KEGG_PATHWAY Pathways in cancer RT 9 1.0 7.2E-3 5.9E-2
KEGG_PATHWAY Dorso-ventral axis formation RT 3 0.3 2.0E-2 1.4E-1
KEGG_PATHWAY Cytokine-cytokine receptor interaction RT 7 0.7 2.7E-2 1.6E-1
KEGG_PATHWAY Cyanoamino acid metabolism RT 2 0.2 6.0E-2 3.1E-1
KEGG_PATHWAY Endocytosis RT 5 0.5 7.8E-2 3.5E-1
D
Lv-NC
Lv-shRNA
Lv-NC
Lv-shRNA
TGFBR1
p-SMAD2 (Ser467)
t-SMAD2
p-SMAD3 (Ser423/425)
t-SMAD3
GAPDH
56kDa
58kDa
58kDa
55kDa
55kDa
36kDa
Huh7
Hep3B
E
Lv-NC
Lv-shRNA
IOD value(Relative to GAPDH)
NOTCH1
JAG1
DTX1
*
F
Lv-NC
Lv-shRNA
IOD value(Relative to GAPDH)
TGFBR1
p-SMAD2
t-SMAD2
p-SMAD3
t-SMAD3
*

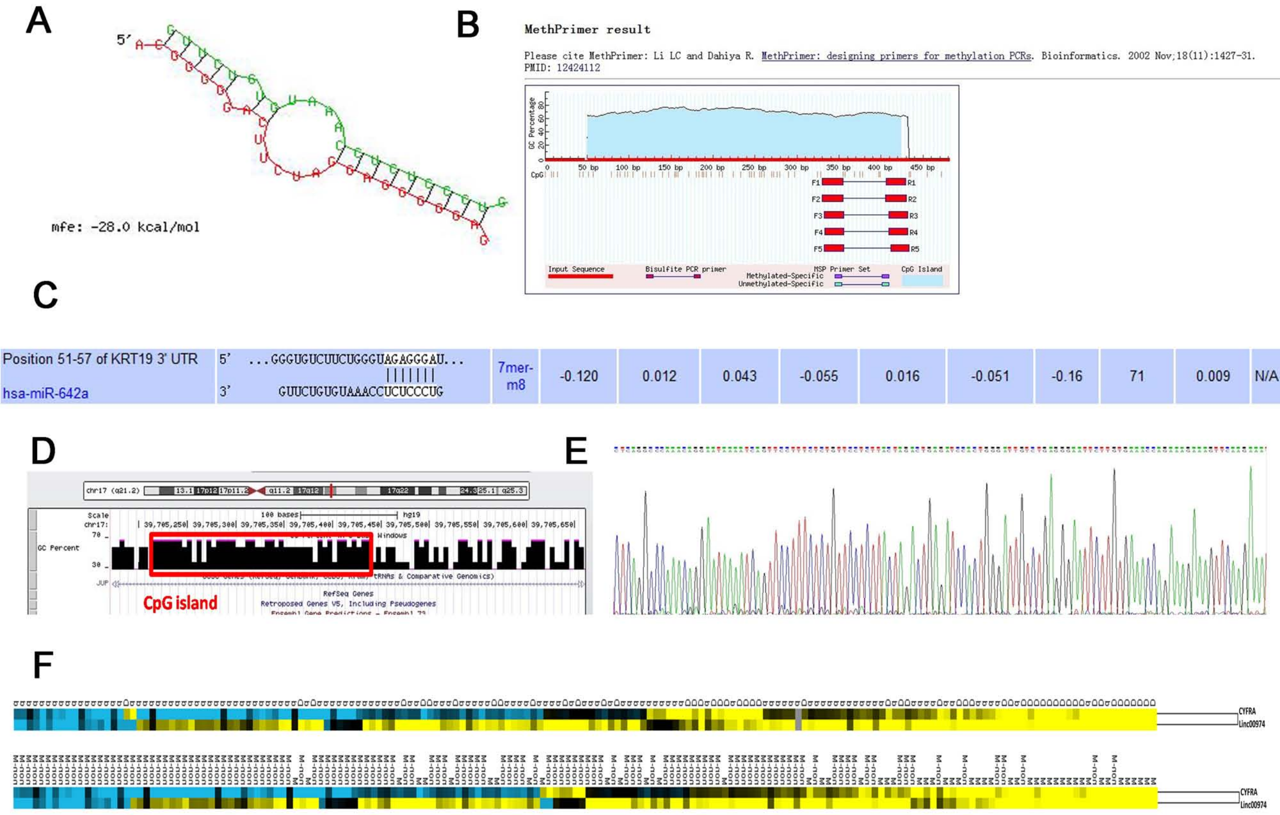
A
5′
mfe: -28.0 kcal/mol
B
MethPrimer result
Please cite MethPrimer: Li LC and Dahiya R. MethPrimer: designing primers for methylation PCRs. Bioinformatics. 2002 Nov;18(11):1427-31.
PMID: 12424112
GC Percentage
CpG
F1 R1
F2 R2
F3 R3
F4 R4
F5 R5
Input Sequence
Bisulfite PCR primer
MSP Primer Set
Methylated-Specific
Unmethylated-Specific
CpG Island
C
Position 51-57 of KRT19 3' UTR
hsa-miR-642a
5' ...GGGUGUCUUCUGGGUAGAGGGAU...
3' GUUCUGUGUAAACCUCUCCCUG
7mer-m8
-0.120
0.012
0.043
-0.055
0.016
-0.051
-0.16
71
0.009
N/A
D
chr17 (q21.2)
Scale
chr17:
100 bases
hg19
GC Percent
CpG island
RefSeq Genes
Retroposed Genes V5, Including Pseudogenes
E
F
CYFRA
Linc00974
CYFRA
Linc00974
